# Mapping Quality Indicators to Assess Older Adult Health and Care in Community-, Continuing-, and Acute-Care Settings: A Systematic Review of Reviews and Guidelines

**DOI:** 10.3390/healthcare12141397

**Published:** 2024-07-12

**Authors:** Mehri Karimi-Dehkordi, Heather M. Hanson, Megan Kennedy, Adrian Wagg

**Affiliations:** 1 Faculty of Medicine & Dentistry, Keyano College, University of Alberta, Edmonton, AB T6G 2R3, Canada; 2 Provincial Seniors Health and Continuing Care, Alberta Health Services, Cumming School of Medicine, University of Calgary, Calgary, AB T2N 1N4, Canada; heather.hanson@albertahealthservices.ca; 3 Geoffrey and Robyn Sperber Health Sciences Library, University of Alberta, Edmonton, AB T6G 2R3, Canada; mrkenned@ualberta.ca; 4 Provincial Seniors Health and Continuing Care, Alberta Health Services, Faculty of Medicine & Dentistry, University of Alberta, Edmonton, AB T6G 2R3, Canada; wagg@ualberta.ca

**Keywords:** quality indicators, older adult health, older adult care, community-care settings, continuing-care settings, acute-care settings

## Abstract

Quality indicators (QIs) play a vital role in enhancing the care of older adults. This study aimed to identify existing QIs relevant to the health and care of older adults in community-care, continuing-care, and acute-care settings, along with available information such as definitions and calculation methods. A systematic review of published review studies, grey literature, and guidelines was undertaken, utilizing six electronic databases searched for materials dated from 2010 to 2 June 2023. To be included in this study, the literature had to provide data on QIs in a setting involving older adults. This study included 27 reviews and 44 grey literature sources, identifying a total of 6391 QIs. The highest number of indicators (37%) were relevant to continuing care; 32% and 28% were pertinent to community- and acute-care settings, respectively. The process domain had the highest number of QIs (3932), while the structure domain had the fewest indicators (521). A total of 39 focus areas were identified, with the five most common areas being, in descending order, orthopedics/hip fractures, end-of-life/palliative care, appropriate prescribing, neurocognitive conditions, and cardiovascular conditions; these areas ranged between 10% and 6%. When mapped against the Quadruple Aim framework, most QIs (85%) were linked to improving health outcomes. This inclusive compilation of QIs serves as a resource for addressing various focus areas pertinent to the Quadruple Aims. However, few quality indicators have been designed to provide a comprehensive and thorough evaluation of a specific aspect, taking into account all three key domains: structure, process, and outcomes. Addressing the description and psychometric properties of QIs is foundational for ensuring their trustworthiness and effective application.

## 1. Introduction

Worldwide, the proportion of older adults in the population continues to rise. From 2015 to 2050, it is expected that, globally, the relative proportion of adults over 60 years old will roughly double, from 12% to 22% [1]. The health states of older adults range from being healthy, free from severe illness, and independent to being dependent and affected by several chronic diseases [2]. For those older adults with higher levels of chronic disease and complex needs, the demand for health care services across the continuum of care is anticipated to escalate [3]. Effective health promotion and disease prevention programs embedded in primary care and public health will be required to reduce acute and chronic disease incidence and prevalence. Given the importance of improving the quality-of-care outcomes and helping older adults, care providers, and policymakers to make informed decisions, healthcare systems need to ensure the effectiveness, equity, and efficiency of services to keep up with the growing demand [4]. In many areas, quality indicators (QIs) are used to measure older adults’ health status and the quality of healthcare service performance [2]. Data from QIs can also contribute to the identification of the areas that require further improvement, with feedback then used to drive improvement [5]. The last few decades have seen a significant increase in the literature on quality indicators pertaining to older adults’ health status and the quality of health care provided to this population; as a result, there are now a large number of quality indicators. Several reviews have identified and collated relevant QIs, but these have been limited in scope. To date, no evidence review has comprehensively synthesised all types of quality indicators related to health conditions for older adults across different healthcare settings. Therefore, the overarching objective of this study was to provide a comprehensive set of structural, process-based, and outcome-based QIs for measuring the quality of care for older adults across the healthcare continuum. Our aims were as follows:

(1) Identify existing QIs relevant to the health and care of older adults in community care, continuing care, and acute care;
(2) Categorize the identified QIs based on key attributes to create a taxonomy to facilitate their selection and application.

## 2. Materials and Methods

The current systematic review was registered on PROSPERO (International prospective register of systematic reviews; CRD42024535523) and was conducted and reported in accordance with the Preferred Reporting Items for Systematic Reviews and Meta-Analyses (PRISMA) checklist (File S1) [6].

### 2.1. Search Strategies, Inclusion and Exclusion Criteria

A systematic review was employed given the plethora of works in the literature and the lack of a synthesized resource in this area. The systematic search was designed in conjunction with a specialized health sciences librarian (MK), aiming to identify any potential types of synthesis review, such as systematic reviews or scoping reviews, or guidelines.

The search strategy included seven electronic databases: Medline, EMBASE, and PsycINFO via OVID; CINAHL via EBSCOhost; Scopus; Cochrane Library via Wiley; and TRIP Pro.. The search strategy was derived from three main concepts: (1) elderly population; (2) aging or health; and (3) quality indicators or benchmarking (File S2).

For the purposes of this study, a “quality indicator” was defined as a quantitative measure of any of the structure-based, process-based, or outcome-based aspects of health care [7]. To be included, articles had to be a type of peer-reviewed review article. The work in the literature had to be in the English language and had to have been published between 2010 and 2 June 2023. Including studies from 2010 onwards enhanced the applicability of findings to current healthcare settings, striking a balance between comprehensiveness and a manageable scope for the review, and facilitating a robust synthesis of evidence as to quality indicators. Additionally, articles had to include at least one quality indicator used to measure any of the four main areas: (a) older adults’ health promotion, disease prevention, or population-based/public health; (b) older adults’ health care in the context of community/primary care, acute care, or continuing care; (c) costs of older adults’ care in acute care or continuing care; or (d) workforce outcomes with relation to older adults’ care in acute care or continuing care.

**Figure 1:**
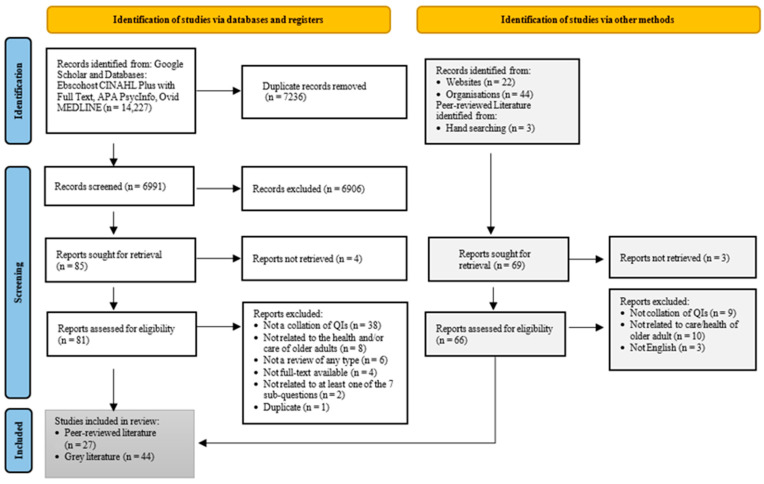
PRISMA flowchart for selection of review studies and grey literature focusing on QIs relevant to the health and care of older adults.

We excluded articles if the focus was not to collate quality indicators but rather to identify, for example, the effectiveness of interventions using QIs; articles were also excluded if the method was not solely a review of the literature but rather a report of a systematic review combined with a Delphi survey. Figure 1 shows the PRISMA flow diagram describing the study selection process.

Title screening was conducted independently (by MKD and HH) using a web-based systematic review manager (Veritas Health Innovation, Melbourne, Australia. Available at www.covidence.org, accessed on 3 November 2020). To enhance the extent to which independent reviewers screened the articles at the title/abstract and full-text stages and reached the same decisions, HH and MKD performed the screening of 145 abstracts and full-text articles in two stages.

Subsequently, MKD reviewed all citations, and HH reviewed 10% of the articles excluded at the title/abstract stage and all citations at the full-text screening. In this stage, the reviewers were in absolute agreement (100%) about which articles to exclude at the title-and-abstract screening stage. For purposes of statistical analysis Cohen’s kappa was calculated to examine inter-rater agreement at the full-text screening. The results revealed a perfect agreement level between the two reviewers (Cohen’s k: 0.83, percent of agreement: 94.2). Discrepancies were resolved by consensus. 

A companion grey-literature search was also executed to obtain additional relevant records. To do so, we first used two databases of grey literature, namely, CADTH (Canadian Agency for Drugs and Technologies in Health), and TRIP Pro. The Google search engine was then used to identify the official websites of national and international governmental organizations, healthcare organizations, and agencies located in Nordic or European countries, North America, Australia, or New Zealand that contained reports and guidelines discussing quality indicators directly addressing seniors’ health. We employed the Joanna Briggs Institute (JBI) critical appraisal assessment checklist for systematic reviews to rate the methodological quality of the included studies [8]. Critical appraisal was undertaken by one reviewer, with questions or concerns brought to the research team as required (Table S1). The grey literature assessed and included in this study met the criteria for currency, relevancy, authority, accuracy, and purpose (CRAAP) in light of the inclusion criteria [9]. 

### 2.2. Data Extraction and Coding

Standardized data extraction tools from the Joanna Briggs Institute informed the construction of our data extraction tool. We also extracted data, if available, about the QIs, clinical domains/subdomains, indicator descriptions, data sources, types of health care, relevant settings, numerators, and denominators. If available, data on the reliability, validity, feasibility, and risk adjustment of the QIs were also extracted.

Analysis was an iterative process of inductive and deductive coding processes. The analysis yielded three main attributes of quality indicators, including setting, type of measures, and focus area. Focus areas were also mapped to the Quadruple Aim, a framework highlighting four primary aims for restructuring healthcare delivery systems, including optimizing the patients’ experience of care, the health of populations, the healthcare provider experience, and value for money [10].

To identify the corresponding settings for QIs, we initially, extracted explicit relevant data from the literature and, in places, found that the operational or theoretical definitions of settings employed across the relevant literature were inconsistent or that information about the setting was absent. Thus, to ensure harmonization of the coding protocol, we conducted focused coding of all initial codes by re-reading information provided about the indicator or its elements (e.g., description, numerator, and denominator) to determine the setting according to the four predefined settings (acute care, community/primary care, continuing care, and the “4 Ps” (prevention, promotion, population, or public health)) (Table 1).

We applied the same method to identify suggested approaches to assessing the quality of care. We first extracted the available data relevant to the types of QIs and found that, although the triad of structure, process, and outcomes (using the Donabedian SPO framework) were used to categorize 41% of the quality indicators, the underpinning theoretical or conceptual framework for such assessments was inconsistent. Therefore, to ensure consistent and coherent coding and determine the most appropriate domain for each indicator, we employed the definitions provided by Donabedian’s model through reading and re-reading the information available for each QI: structure-based refers to the situations under which care is delivered; process-based refers to the actions which comprise health care; and outcome-based refers to desired or undesired changes, in individuals and populations, attributable to health care [11]. Thus, each quality indicator was consistently attributed to one of Donabedian’s three domains.

To assign a focus area for each quality indicator, the first-level coding was carried out by examining the data relevant to QIs found in the peer-reviewed literature. We then developed a codebook containing the key focus areas and operational definitions to ensure objectivity and reliability during the coding process, with questions being resolved via consensus discussion. The focus areas, accompanied by their definition and two example Qis, can be found in Table 2.

As a taxonomy, the codebook was a guide for data management and interpretation which evolved through the iterative analysis process. The focus area of each indicator was assigned based on the main focus of the studies (e.g., cardiovascular conditions) and/or the information provided in quality measurement components, including the indicator’s name, description, nominator, and denominator. For example, “End of Life/Palliative Care” was determined to be the focus area for an indicator which was described as “discussion of the strategy of care among physicians and nurses,” with the denominator of “all patients who died”. 

This hybrid approach was also used to map the quality indicators to the four equally weighted dimensions of care of the Quadruple Aim framework [10], a broadly accepted guide to enhancing the quality of health system performance. The four aims consist of improving population health, optimizing the patient’s experience, ensuring sustainability (reducing costs), and enhancing the provider’s experience. Quality indicators focusing on the disease-specific measures (e.g., cardiovascular illness and depression) or generic health-related measures (e.g., physical functioning and pain) were mapped to the population health domain. Quality indicators addressing the patient’s or caregiver’s experience of care as subjective (e.g., satisfaction with care) or objective (e.g., receives a follow-up call within 48 h after hospital discharge) measures were mapped to the goal addressing the patients’ experience. Quality indicators measuring aspects of costs and health-services provision were mapped to sustainability. Quality indicators measuring clinical workforce experience and conditions and practice satisfaction were mapped to the provider-based aim. 

Quality indicators were heterogenous in form; while some were presented in a structured fashion, such as the if–then statement or explanatory/measurement statement, others were less structured or brief, either (1) without a measurement statement, or (2) with a measurement statement in either the description or in the objectives of the quality indicator. Thus, to maintain structural consistency, we selected the measurement statement (e.g., the percentage of falls) as the quality indicator when this was available. 

We established two characteristics relevant to the minimum threshold for identifying QIs with a determinate degree of quality: clarity (the QI must be sufficiently clear to facilitate meaningful interpretation and action) and quantifiability (the QI must be quantifiable and measurable accurately across different contexts and time periods). This requires the explanation of the numerator and denominator to enable proper calculation. Adherence to both criteria should allow operationalization of the QI. 

We compiled all the information into an Excel spreadsheet with two tabs: one for all QIs and another for QIs that met the minimum threshold.

## 3. Results

A total of 27 reviews were identified among the 14,225 articles retrieved from the database search and Google Scholar search. Based on the review typology outlined by Sutton et al. (2019) [12], there were six scoping reviews: one review categorized as a literature review, one specified as a critical literature review, and one labeled as a rapid review. The majority of the articles fell under the classification of systematic reviews, a category containing 17 reviews. The grey-literature searches identified 44 additional sources; 13 were online resources, and 31 were published by institutions such as government health departments, professional organizations, provincial agencies, intergovernmental organizations, and intercontinental councils. The accounts took a variety of forms, such as policy and guideline manuals, clinical practice guidelines, standard sets, reference guides, research and survey reports, update reports, governmental/annual reports, government health system news, fact directories, evidence-based recommendations, toolkits, indicator libraries, and strategic plans.

This section comprehensively presents the study’s findings across various dimensions, including detailed analyses of the studies’ characteristics, the characteristics of the QIs, and the distribution of QIs across specific settings. Additionally, this section explores the domains to which QIs are attributed and their focus areas. It also discusses how these findings align with the Quadruple Aims. Furthermore, the section addresses all QIs identified through this study, including those with minimum thresholds.

### 3.1. Study Characteristics

Table S1 presents the quality assessments of the included reviews, ranging from moderate to high. The most prevalent shortcomings, ranked from higher to lower frequency, were identified in question 6, concerning the independent conducting of critical appraisal by reviewers; question 5, regarding the appropriateness of the appraising studies; and question 9, addressing the assessment of the likelihood of publication bias. Question 8, concerning the methods used to combine studies, was deemed not applicable to the majority of the articles, due to their focus on descriptive and mapping analyses. Notably, six scoping reviews and five systematic reviews did not employ a critical appraisal tool and three were insufficiently clear in doing so. Among those that did, three systematic reviews utilized tools such as QUALIFY and CASP, while 10 reviews used tools such as ARIE (Appraisal of Indicators through Research and Evaluation) to appraise the psychometric properties of the QIs. 

### 3.2. Quality Indicators’ Characteristics

A total of 6391 QIs were identified (Table 3), following the removal of 191 duplicates (3%). Of these, 29% of the duplications related to end of life/palliative care, followed by nutrition (16%), elimination conditions (9%), workforce (6%), and functional abilities (6%), respectively. The detailed information about the characteristics of the 6391 QIs is presented in Supplementary Material Excel S1 (Tab 1).

Analysis of some of the essential indicator components was conducted to understand indicator quality and completeness. The description of the QI was stated for 52% of the indicators. The measurement method, including the description of the numerator, denominator and its calculation, was presented for 39% of the indicators. Information about validity and reliability was presented for 11% and 3.4% of the QIs, respectively. The assessment tools or data sources were stated for 56% of the indicators. Performance standards usable as the criteria or benchmark were provided for the interpretation of the results of 8% of the QIs, in at least one of three ways, by providing (1) a numeric criterion (e.g., volumes above 400 are desirable), (2) a vague statement (e.g., more is better), or (3) an explanatory statement to interpret an indicator when the results are equal to or higher or lower than a point of reference, or when the indicator is complex and multifaceted (e.g., length of stay). 

#### 3.2.1. Quality Indicators by Specific Setting

**Figure 2:**
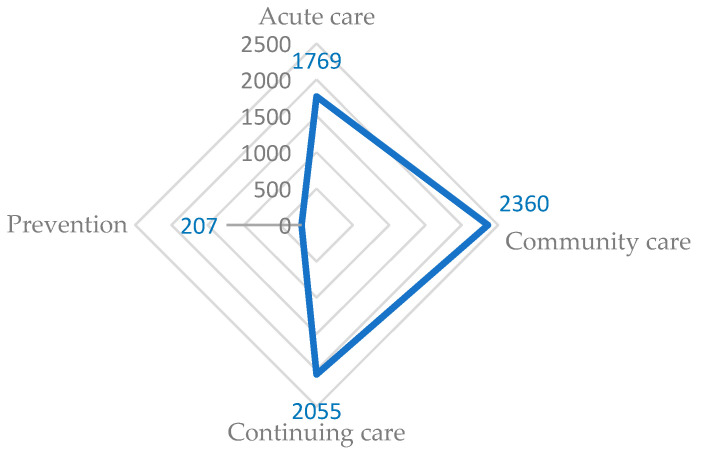
Radar graph showing the proportion of quality indicators assigned to setting.

The majority (37%) of the indicators were relevant to continuing care; 32% and 28% of indicators were pertinent to community- and acute-care settings, respectively (Figure 2). Few indicators corresponded to the 4 Ps (4%). 

#### 3.2.2. Domains Attributed to Quality Indicators 

**Figure 3:**
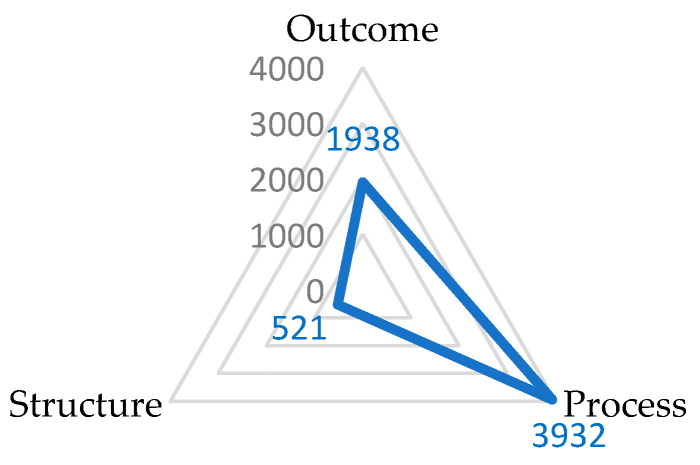
Radar graph showing the proportion of quality indicators assigned to the Donabedian domains framework.

Analysis of the distribution of QIs within the Donabedian classification revealed the magnitudes of difference between the structure-based, process-based, and outcome-based indicators (Figure 3). Most QIs were related to process (n = 3932), and generally addressed the assessment of various aspects of the care plan and were associated with care provider performance (e.g., average time of visits and health assessment). Outcome was the second-most-frequently occurring domain (n = 1938), and contributed most to patients’ mental, social, and physical health outcomes and experience (e.g., a decline in bladder continence, and satisfaction). The structure domain included 521 indicators which predominately concerned the accessibility of resources and the contextual conditions within which care delivery occurred (e.g., costs, policies, and staffing hours).

#### 3.2.3. Quality Indicator Focus Area

**Figure 4:**
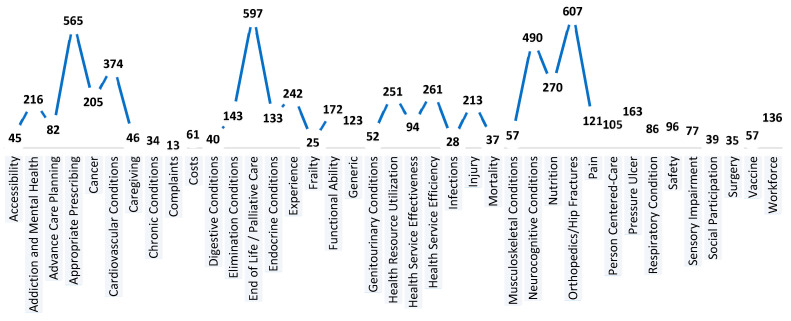
Frequency of indicators categorized within each focus area.

The identified focus areas include both objective and subjective measures. Objective focus areas are based on quantifiable data, such as costs and orthopedics/hip fracture metrics. Conversely, subjective focus areas encompass client-reported outcomes, capturing personal experiences and perceptions. The analysis of the QIs by focus area revealed additional information on which to base classification, namely, generic and disease/context-specific factors. In total, 39 focus areas were identified. The five most frequent areas were orthopedics/hip fractures (10%), end of life/palliative care (9%), appropriate prescribing (9%), neurocognitive conditions (8%), and cardiovascular conditions (6%) (Figure 4). Conversely, the five areas that received least focus were surgery (0.6%), chronic conditions (0.5%), infections (0.4%), frailty (0.4%), and complaints (0.2%). Table 3 displays the taxonomy breakdown by focus area and setting and Quadruple Aims mapping. 

#### 3.2.4. Embracing the Quadruple Aims

**Figure 5:**
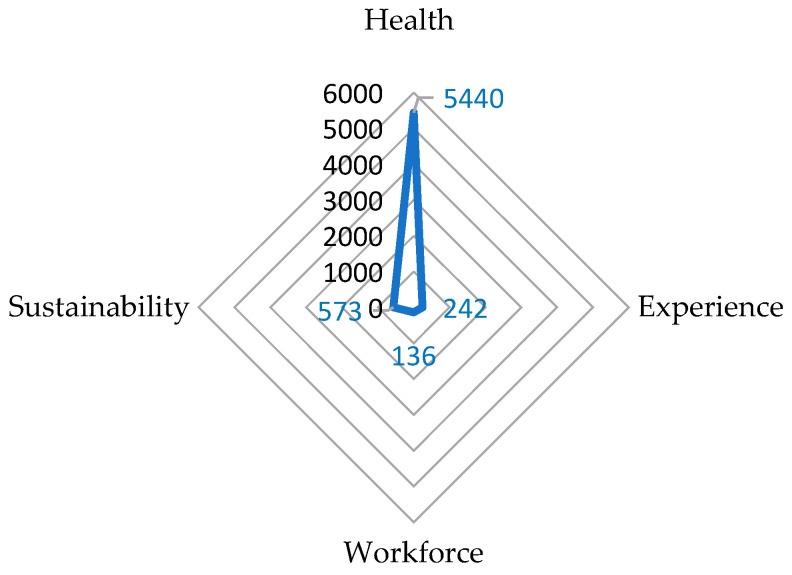
Radar graph showing the proportion of quality indicators assigned to the Quadruple Aim framework.

Mapping against the Quadruple Aims (Figure 5) revealed that 85% of the QIs were related to improving health outcomes, with 34 focus areas applicable to community/primary care (34%), continuing care (25%), acute care (23%), and 4 Ps health (3%). Four percent of the QIs were assigned to the patient experience domain, applicable in various settings, including acute care (0.7%), community/primary care (1.3%), continuing care (2.0%), and 4 Ps health (0.03%). Two percent of the QIs were mapped to the enhancing-provider-experience/workforce component. Here, most QIs were relevant to continuing care (1.2%); the rest were almost equally distributed between acute care and community/primary care settings and 4 Ps health, which accounted for about 0.5%, 0.3%, and 0.3%, respectively. Nine percent of the QIs were assigned to the sustainability domain, and these included three focus areas: health service efficiency, health resource utilization, and costs. QIs related to sustainability were most applicable to acute-care (3.7%), community-care (1.7%), and continuing-care (3.5%) settings, with few being categorized under 4 Ps health (0.1%).

### 3.3. Quality Indicators with Minimum Threshold: Description and Calculation

A significant portion of QIs fail to meet the two minimal standards of clarity and quantifiability. Out of 6391 QIs assessed, only 1662 QIs met both criteria. The list of QIs meeting the minimum threshold is presented in Supplementary Material Excel S1 (Tab 2). Additionally, it is worth highlighting that this category of QIs spans across all 39 assigned focus areas, ensuring comprehensive coverage and holistic evaluation.

## 4. Discussion

A total of 6391 QIs were identified, which were associated with 39 focus areas, of which the focus area of “end of life/palliative care” comprised the highest number. Approximately 3% of QIs were duplicates, indicating that specific QIs—end of life/palliative care, nutrition, elimination conditions, workforce, and functional abilities, in that order—were more commonly reported compared to others. The indicators were applicable across the healthcare continuum or related to prevention/promotion/population/public health. Most indicators were specific to community/primary care. While in most countries, a minority of the population aged 65 and over resides in a congregated-living facility [13], over one-third of identified QIs were aligned with this setting, with slightly fewer relating to community settings, or to acute care. Few of the QIs were devoted to prevention/promotion/population/public health.

The mapping of the indicators against the three Donabedian domains was not straightforward. There was lack of information and clarity in some QIs, which posed a significant challenge in understanding (1) how the indicators can be measured, (2) by whom the action is carried out, (3) whether the QI is meant to measure changes, and (4) from whose perspective the indicator was based, that of clients (e.g., patients, caregivers, or family), the healthcare professionals, or the system? The subjective or objective nature of the indicators also varied widely, perhaps unsurprisingly so, given the variety of conditions and QI domains. By integrating both types of QIs, we achieved a comprehensive evaluation of care quality, addressing both measurable performance and the subjective well-being of older adults.

Thus, when it comes to the development of QIs, the current work emphasizes the need to standardize the expression of indicators in a clear and transparent manner, allowing informed selection and use. This need for clarity aligns with the insights provided by Camp and Cheung (2018) [14], who argued that certain challenges were related to the lack of consensus on a standardized approach for formulating indicator statements. This issue hinders the comparability of work across diverse jurisdictions and also affects the applicability of QIs in healthcare evaluation and improvement. It is notable that only 26% of QIs met two main aspects of quality criteria, to be easily understandable and accurately measurable, and could therefore be categorised among those with this minimal threshold.

The domains of structure, process, and outcome were assigned by the individual citation authors in fewer than half (41%) of the indicators. This appears to have been the case either by merely addressing the domains with no clear interrelationship, or addressing a group of relevant indicators across the three domains to support a comprehensive assessment of the quality of an aspect of care. The latter form, the less common, was more aligned with the tenets of the SPO framework, which emphasizes a combination of efforts to consider all relevant aspects contributing to the quality of care. This comprehensive approach has the potential to identify the causes of failures and determine whether the method of assessment is appropriate [11]. Thus, proper use of the SPO framework deserves special attention when it comes to the development of QIs.

A breakdown of identified QIs into the constructs of the SPO framework revealed a large imbalance in the number of QIs relevant to each of the three domains. More than 60% of the QIs were categorized under process, followed by 30% covering outcome, and almost 10% covering structure, respectively. Two previous systematic reviews, in the context of palliative and oncology care, reported similar findings, indicating that most of the focus was on QIs related to process [15,16]. The imbalance in the use of process indicators and outcome indicators has been related to the complexity and difficulty of employing outcome indicators in some contexts [16], given that outcome measurements can be influenced by a combination of factors, as well as being affected by uncontrollable factors [17]. Other justifications for not focusing on the outcome domain were related to the difficulty of accessing the pertinent information [18], and the challenges associated with the reliability of interpretation, which requires standardization of data collection and adjustment for case-mix [19].

We employed a generic approach, relating QIs to key attributes to create a taxonomy which can serve as a supportive resource for the field. 

This review adds to previously published systematic reviews which focused on a single disease area (e.g., aortic aneurysm and hip fracture), population (e.g., vulnerable elderly persons), or setting. By including both systematic reviews and the grey literature, we captured a complementary landscape of Qis, as for some areas, the scope of the grey literature and the peer-reviewed literature differed, and the two data sources revealed the breadth and multifaceted nature of QIs related to older person’s health. 

Although the peer-reviewed and grey-literature studies included were mostly ranked as being of moderate quality, there was a lack of quality appraisal for the individual QIs, regardless of source. This was echoed in the quality of data available for each individual quality indicator, including description, numerator, denominator, strategy for risk adjustment, validity, reliability, data sources, and performance standard. The extent of the information provided with the indicators varied remarkably. This is a considerable limitation in the quality of the indicators, since the process of the selection of indicators and the availability of detailed information can influence data collection and computation and the interpretation of the data. This area requires further attention as to indicator development. This gap aligns well with the findings of existing studies, which reported that despite many QIs being found, few indicators were described with a level of detail sufficient to accurately examine the quality of care [16]. This gap exists across focus areas. 

Based on this systematic review, recommendations for developing QIs include clearly defining each indicator by including the target population, measure, focus area, and time-frame, in order to determine whether it is for the measurement of a specific aspect (e.g., risk of falls); clearly define for which Donabedian domain it is intended; and provide sufficient detailed descriptive information, including field, settings, data source, psychometric properties (e.g., validity, reliability, and feasibility) and calculation details (e.g., numerator, denominator, and the range for interpretation of results) to ensure its further use. 

As with most studies, there are limitations to our work. In our preliminary coding, ‘Indigenous’ emerged as a population-based focus area for relevant indicators. However, indicators did not suggest linguistic and cultural sensitivity to the indigenous population, and thus those indicators were re-categorized into the relevant condition area rather than the intended population; this may be an unintended limitation of this work. Further research is warranted to address population-sensitive indicators. In addition, we did not identify studies regarding pandemic-related indicators, which may present an area for future research. 

## 5. Conclusions

This systematic and grey-literature review has produced a valuable taxonomy of the landscape of QIs used to measure quality of care for older adults. Producers and users of QIs can identify content of relevance based on setting, focus area, and/or domain, which can accelerate the refinement and application of QIs. As such, the list of identified QIs forms a useful resource to support quality-of-care for older adults across the healthcare continuum. The comprehensive list of QIs meeting the established minimum threshold provided in Excel S1, Tab 2, allows potential users to select a QI for evaluation and utilization in quality-improvement endeavors. The significance of this study extends beyond providing a mere list; it forms a practical guide for clinical practice and health policy formulation. Rather than generating new standalone QIs, by selecting QIs which are well-defined, characterised by clear measurement, and validated by psychometric properties, clinicians can identify critical areas for intervention, ensuring that care is both effective and patient-centered. Policymakers can select and leverage these QIs to establish standardized benchmarks, promoting consistency and excellence in healthcare delivery. Furthermore, QIs can drive continuous improvement in healthcare services, facilitate the adoption of best practices, and ultimately enhance the health outcomes and quality of life for older adults. The study, therefore, stands as a foundational reference for both improving clinical practices and informing robust health policies.

## Figures and Tables

**Table 1:** Predefined concepts in the inclusion criteria.

Concept	Operational Description
Older Adults	Older adults were defined as “individuals 65 years of age and over.” Other terms included “aged”, “elderly”, and “seniors”. Indicators covering a broad range of ages were also included if age adjustment was used.
Quality Indicators	The article presented at least one quality indicator which focused on health care; health outcomes; caregiver’s health outcome; care experience; or the caregivers’ care experience, workforce, or finances.
Review Articles	Any type of review, including, but not limited to, qualitative, quantitative, or mixed-methods systematic reviews; meta-analyses; meta-ethnography reviews; narrative reviews; meta-narrative reviews; update reviews; reviews of psychometric properties; umbrella reviews; literature reviews; integrative reviews; scoping reviews; critical reviews; Cochrane reviews; mapping reviews; or rapid reviews.
Settings/Contexts	Quality indicators were related to the following settings or contexts: (1) The 4 Ps (prevention, promotion, population, or public health) were used to indicate the prevention of disease or illness, health promotion, or population-based/public health measures. (2) Community or primary care referred to community health and primary healthcare. (3) Acute care was used to indicate an emergency or acute medical intervention and care, including emergency-related dispatch services, care, transport, and hospital care. (4) Continuing care was used to indicate home care, supported living (assisted living), or long-term care (nursing homes).

**Table 2:** Descriptions of focus area and examples.

Focus Area	Practical Definition	Examples of Indicator
Accessibility	Relating to the ability to access information or care in a reasonable mode/language/setting/time, or within a reasonable distance, or equitable opportunities to receive necessary health services or outcomes	▫ Percentage of patients who report being able to see a general practitioner (GP)/nurse practitioner (NP) on the same day or next day; ▫ Information delivered in multilanguage format.
Addiction and Mental Health	Relating to addictive behaviours and/or emotional or psychological wellbeing	▫ Percentage of residents who have become more depressed or anxious; ▫ If a vulnerable elder uses tobacco regularly, then he or she should, at least once, be offered counseling or pharmacologic therapy to stop tobacco use.
Advance Care Planning	Relating to the providing of instructions for future care decisions	▫ All vulnerable elders should have, noted in the outpatient chart, an individual surrogate decision-maker or documentation of a discussion aiming to identify or search for a surrogate decision-maker; ▫ Percentage of patients with documentation of resuscitation status.
Appropriate Prescribing	Relating to the avoidance of inappropriate medications, use of medications as indicated, and attention to side effects/risk–benefit ratio.	▫ All vulnerable elders should have an annual drug-regimen review; ▫ Prevalence of antipsychotic drug use in the absence of psychotic and related conditions.
Cancer	Relating to the diagnosis or treatment of cancer	▫ If a cancer patient is admitted to a hospital, then there should be screening for the presence or absence of pain; ▫ Palliative care should be accessible to all patients and families in association with a cancer diagnosis, in a timely manner, throughout the entire duration of their disease.
Cardiovascular Conditions	Relating to treatments or diseases of the cardiovascular system, such as those affecting the heart and blood vessels	▫ All patients with a diagnosed coronary artery disease should be prescribed aspirin 75–150 mg/day unless contraindicated; ▫ Aneurysm-related death.
Caregiving	Relating to the activity of regularly supporting an older adult spouse/parent/family member/friend/neighbour	▫ Percentage of informal caregivers reporting levels of stress, with response options including extremely stressful, quite a bit stressful, a bit stressful, not very stressful, and not at all stressful; ▫ Hours (number/mean) of care per week by informal caregiver.
Chronic Conditions	Relating to the treatment/management of conditions or diseases that require ongoing medical care	▫ Evidence of a locally coordinated approach intended to ensure that older people with multiple long-term conditions having a care and support needs assessment have their physical and mental health needs included; ▫ Unplanned hospitalisation for chronic ambulatory care-sensitive conditions.
Complaints	Expressions regarding unsatisfactory or unacceptable events/occurrences and the associated actions taken to address the issue	▫ Presence of an access point, an office devoted to receiving and collecting complaints, signals, suggestions, by everyone (yes, no); ▫ Percentage of actions taken by a provider in response to a complaint, by action category.
Costs	Relating to the financial expenditures associated with healthcare services or delivery	▫ Consultation skipped due to costs; ▫ Proportions of cost for acute, residential, community health, and primary care.
Digestive Conditions	Relating to the intake and processing of foods, including the stomach, liver, and gallbladder (not including the pancreas, see Endocrine Conditions)	▫ Proportion of 65–74 year-olds and adults 75 years and older who have lost all their natural teeth; ▫ If a patient with advanced end-stage liver disease who is not a candidate for transplant has new hemodialysis or placement of a pacemaker or implantable cardioverter-defibrillator (ICD), then within one month prior to the procedure, the medical record should document the goals of care and the patient’s preference for the intervention, because a patient’s treatment should reflect his or her goals of care.
Elimination Conditions	Relating to the excretion of wastes by bladder or bowel, including continence, catheterization, and urinary-tract infections and treatments	▫ Percentage of long-stay residents who have had an indwelling catheter in the past 7 days; ▫ Residents whose bladder continence worsened.
End of life/Palliative care	Relating to terminal stage care and/or management of symptoms rather than cure	▫ The percentage of palliative/end of life patients who died in their preferred place of death; ▫ Document a comprehensive and holistic assessment of palliative-care needs among patients with progressive, life-limiting illnesses who have been identified as able to benefit from palliative care (Primary Care).
Endocrine Conditions	Relating to conditions/diseases of the endocrine glands, including the pancreas	▫ If a vulnerable elder has an elevated glycosylated hemoglobin level, then he or she should be offered a therapeutic intervention aimed at improving glycemic control within 3 months if the glycosylated hemoglobin level is 9.0% to 10.9%, and within 1 month if the glycosylated hemoglobin level is 11% or greater; ▫ Uncontrolled-diabetes admission rate.
Experience	Relating to patient/client/resident satisfaction or the experience of receiving care/service	▫ Proportion of people feeling supported in managing their conditions; ▫ The percentage of discharged patients who responded positively to the following question: Did you receive enough information from hospital staff about what to do if you were worried about your condition or treatment after you left the hospital?
Frailty	Relating to a state of increased vulnerability or decline (including in/dependence), in two or more domains	▫ Proportion of older people most at risk of a decline in their independence and mental wellbeing who take part in tailored, community-based physical activity programmes; ▫ If a vulnerable elder is admitted to a hospital or is new to a physician practice, then multidimensional assessment of cognitive ability and assessment of functional status should be documented.
Functional Ability	Relating to the performance of tasks of normal life, including ADLs, IADLs, transferring, mobility, balance, and gait	▫ Percent of residents who have had an unexpected loss of function in some basic daily activities; ▫ Percentage of short-stay residents who were discharged and gained more independence in transfer, locomotion and walking during their episodes of care.
Generic	Relating to broad focus; relevant for most patients or settings	▫ Numbers and percentages of those over 65 and 75 living in housing with poor or extremely poor amenities; ▫ Average assistance time.
Genitourinary Conditions	Relating to conditions or diseases of the urinary or reproductive systems	▫ If a male vulnerable elder patient complains of new lower urinary tract symptoms (LUTS), then a rectal examination (including prostate size, degree of tenderness, and nodularity) and abdominal examination should be performed; ▫ Urinary tract infection admission rate.
Health Equity	Relating to fair or equal opportunities to receive the necessary health services or outcomes given efforts to address avoidable differences based on population groups	▫ Equity of access to dental care services; ▫ Access to care, i.e., fair and equitable access to care for patients and families, regardless of financial considerations, indicates a good quality of care.
Health Resource Utilization	Relating to the use (overuse or underuse) of services, facilities, or available resources	▫ The median number of days patients remain in hospital when no longer requiring it until home-care services or supports are ready; ▫ Rate of total hospital emergency department (ED) visits per 1000 patients.
Health Service Effectiveness	Relating to achieving the desired outcome of a healthcare service or practice	▫ If the elements of a comprehensive geriatric assessment are performed, then follow-up should assure the implementation of recommendations; ▫ The proportion of home-care services in which a review was undertaken at least every two months.
Health Service Efficiency	Relating to the optimal use of resources	▫ The percentage of the population that reported having a regular health care provider; ▫ Average waiting time for access to care accommodation and nursing home.
Infections	Relating to general invasion of a microorganism such as bacteria or viruses (system-specific infections are excluded, i.e., pneumonia)	▫ The number of Clostridium difficile infections (C-diff) acquired in hospital per 10,000 patients’ days; ▫ If a hospitalized vulnerable elder patient has a new fever (body temperature, 38.5 °C [101.3 °F]), then there should be documentation that a physician’s examination was performed within 4 h (or fever evaluation performed in the last 48 h, or an alternative explanation for the fever documented in the chart).
Injury	Relating to damage resulting from an external force	▫ Percent of long-stay residents who have experienced one or more falls with a major injury in the target or look-back period; ▫ Newly admitted LTC residents assessed for fall risks.
Mortality	Relating to deaths within a population	▫ The ratio of actual number of deaths compared to the expected number of deaths based upon the types of patients admitted to hospitals; ▫ Potential years of life lost prematurely due to all causes, per 100,000 people.
Musculoskeletal Conditions	Relating to the diagnosis, treatment, or outcomes of diseases of the musculoskeletal system, such as arthritis and joint or connective tissue conditions or diseases (excluding osteoporosis or orthopaedics/hip fractures)	▫ Percentage of residents with contractures in nursing home; ▫ If a vulnerable elder has symptomatic osteoarthritis (OA) and has difficulty with the ambulatory activities of daily living, then the need for activity-of-daily-living assistive devices should be assessed.
Neurocognitive Conditions	Relating to conditions or diseases of the brain affecting cognition, such as dementia, delirium, or learning disabilities	▫ If a patient has advanced stage dementia, then his performance in swallowing and his positioning should be evaluated; ▫ Evidence of local arrangements to ensure that GP practices offer annual health checks for people with a learning disability as they grow older.
Nutrition	Relating to food and/or nutrient consumption for optimal health	▫ Percentage of residents who eat less than 50% of their meals and receive less than one min of assistance; ▫ If a vulnerable elder patient has involuntary weight loss of ≥10% of body weight in ≤1 year, then weight loss (or a related disorder) should be documented in the medical record in recognition of undernutrition as a potential problem.
Orthopedics/Hip Fractures	Relating to the diagnosis and/or treatment of diseases or injuries of the skeletal system, such as hip fractures	▫ If an ambulatory vulnerable elder patient has an osteoporotic fracture diagnosed, then physical therapy or an exercise program should be offered within 3 months; ▫ The number of hours patients waited, from the time of first registration in an emergency department (ED) with a hip fracture (index admission) to the time that patients received hip-fracture repair surgery.
Pain	Relating to the occurrence or treatment of painful sensations	▫ Percentage of all patients with a documentation-of-pain assessment within 48 h of admission; ▫ Percentage of residents whose pain worsened since the prior assessment.
Person-centered Care	Relating to the involvement of patients/clients/residents in their own care and decision-making to ensure personalized care	▫ Percentage of patients and clients who were always or often involved in the care decisions when they saw their doctor or nurse practitioner; ▫ The proportion of home-care services, with space on form for client preferences.
Pressure Ulcer	Relating to the formation or treatment of a break of the skin/tissue	▫ Prevalence of residents’ pressure ulcers (stages 1–4); ▫ If a vulnerable elder patient is at risk of, or suffering from, decubitus, then the general practitioner should consult a dietician.
Respiratory Conditions	Relating to conditions or diseases of the respiratory system, including asthma, COPD, and pneumonia	▫ If a vulnerable elder patient with community-acquired pneumonia is to be discharged home, then the patient should not be unstable on the day before or the day of discharge; ▫ If a vulnerable elder patient has daytime sleepiness and observed apneas or loud snoring, then he or she should be referred for sleep evaluation within 6 months.
Safety	Relating to harmful, unintended, or unfavourable result of a disease, treatment, or intervention	▫ There is a documented procedure to analyse and follow-up on adverse events; ▫ If a patient aged 65 years or older is discharged from the ED to home or residential accommodation, then a risk assessment is required to be performed and documented prior to discharge from the ED.
Sensory Conditions	Relating to conditions of the sensory systems, including sight, hearing, and proprioception	▫ All vulnerable elder patients should have an annual evaluation of hearing status; ▫ If a vulnerable elder patient has new-onset eye pain, grossly visible corneal lesions, or severe purulent discharge, then he or she should undergo a basic eye examination within 72 h.
Surgery	Relating to operative procedures and post-operative outcomes of surgical specialties, excluding orthopaedic surgery	▫ Percentage of surgeries in which a surgical safety checklist was used; ▫ Postoperative wound dehiscence rate.
Vaccine	Relating to vaccinations to prevent infection by virus	▫ If pneumococcal or influenza vaccination rates among patients of a health delivery organization are low (60% of persons at risk for pneumococcal and influenza disease and, 90% of institutionalized elderly patients), then methods to increase the rate of vaccination should be used; ▫ Influenza vaccination rate in the elderly population.
Workforce	Relating to the numbers, safety, and workplace experience of the health system workforce	▫ All team members have certified (accredited?) training in palliative care, as appropriate to their discipline; ▫ The number of lost-time injury claims per 100 full-time equivalent workers in the health care sector per year.

**Table 3:** Frequency of the quality indicators based on the Quadruple Aims, focus areas, and settings.

		Settings					
Quadruple Aim	Focus Area	Acute Care	Community/ Primary Care	Continuing Care	Prevention/Promotion/ Population/ Public Health	Total	Percent
Aim 1: Improving Population Health							
	Accessibility	12	20	12	1	45	1%
	Addiction and Mental Health	19	100	74	23	216	3%
	Advance Care Planning	64	12	6	0	82	1%
	Appropriate Prescribing	100	312	145	8	565	9%
	Cancer	70	106	2	27	205	3%
	Cardiovascular Conditions	166	185	17	6	374	6%
	Caregiving	3	15	27	1	46	1%
	Chronic Conditions	4	25	1	4	34	1%
	Complaints	3	0	10	0	13	0%
	Digestive Conditions	19	4	10	7	40	1%
	Elimination Conditions	13	52	78	0	143	2%
	End of life/Palliative care	253	123	221	0	597	9%
	Endocrine Conditions	15	108	9	1	133	2%
	Frailty	2	9	12	2	25	0%
	Functional Ability	12	52	105	3	172	3%
	Generic	5	20	65	33	123	2%
	Genitourinary Conditions	4	41	7	0	52	1%
	Health Service Effectiveness	26	54	12	2	94	1%
	Infections	13	2	14	0	28	0%
	Injury	50	77	80	6	213	3%
	Mortality	10	1	19	7	37	1%
	Musculoskeletal Conditions	1	42	9	5	57	1%
	Neurocognitive Conditions	82	243	161	4	490	8%
	Nutrition	33	46	183	8	270	4%
	Orthopedics/Hip Fractures	312	284	8	3	607	10%
	Pain	23	34	64	0	121	2%
	Person-Centered Care	15	23	66	1	105	2%
	Pressure Ulcer	43	25	94	1	163	3%
	Respiratory Condition	40	32	12	2	86	1%
	Safety	17	11	66	2	96	2%
	Sensory Impairment	8	57	8	4	77	1%
	Social Participation	2	6	20	11	39	1%
	Surgery	29	5	0	0	35	1%
	Vaccine	4	21	18	14	57	1%
Aim 2: Optimizing the Patient Experience							
	Experience	43	79	118	2	242	4%
Aim 3: Enhancing Provider Experience							
	Workforce	20	24	78	14	136	2%
Aim 4: Ensuring Sustainability							
	Costs	2	21	34	4	61	1%
	Health Resource Utilization	165	31	55	0	251	4%
	Health Service Efficiency	67	58	135	1	261	4%
Total		1769	2360	2055	207	6391	100%

## Data Availability

Data are contained within the article and Supplementary Materials.

## Supplementary Materials

The following supporting information can be downloaded at: https://www.mdpi.com/article/10.3390/healthcare12141397/s1, File S1: PRISMA checklist; File S2: Search Strategies; Table S1: The methodological quality assessment of the included studies; Excel Table S1: Quality indicators for the health and care of older adults. References [20,21,22,23,24,25,26,27,28,29,30,31,32,33,34,35,36,37,38,39,40,41,42,43,44,45,46,47,48,49,50,51,52,53,54,55,56,57,58,59,60,61,62,63,64,65,66,67,68,69,70,71,72,73,74,75,76,77,78,79,80,81,82,83,84,85,86,87] are cited in the Supplementary Materials.
