# Supplementary material for: Mapping Quality Indicators to Assess Older Adult Health and Care in Community-, Continuing-, and Acute-Care Settings: A Systematic Review of Reviews and Guidelines
Source: Healthcare (Basel). 2024 Jul 12;12(14):1397. doi: 10.3390/healthcare12141397 (PMC11276513; doi:10.3390/healthcare12141397)
Supplement: Supplementary file 1 [file healthcare-12-01397-s001.zip › File S2. Search strategies..pdf]

File S2. Search strategies.

Preliminary Citations Retrieved from Databases and Google Scholars

Ovid MEDLINE(R) ALL <1946 to June 2023>

#	**Searches**
1	exp Quality Indicators, Health Care/
2	Functional assessment.mp.
3	Program Evaluation/
4	(project adj1 (evaluat* or effective*)).ti,ab.
5	((quality or performance or process or outcome*) and indicator*).mp. [mp=title, abstract, original title, name of substance word, subject heading word, floating sub-heading word, keyword heading word, organism supplementary concept word, protocol supplementary concept word, rare disease supplementary concept word, unique identifier, synonyms]
6	(program* adj1 (evaluat* or effective*)).mp. [mp=title, abstract, original title, name of substance word, subject heading word, floating sub-heading word, keyword heading word, organism supplementary concept word, protocol supplementary concept word, rare disease supplementary concept word, unique identifier, synonyms]
7	((quality or performance or process or outcome*) adj1 assessment*).ti,ab.
8	bench marks.ti,ab.
9	1 or 2 or 3 or 4 or 5 or 6 or 7 or 8
10	exp Long-Term Care/ or exp Nursing Homes/
11	Continuing care.mp.
12	10 or 11
13	exp Aged/
14	Seniors.ti,ab.
15	13 or 14
16	Hospitalization/
17	Hip Fractures/
18	EMERGENCY SERVICE, HOSPITAL/
19	Acute Care for Elders.ti,ab.
20	acute medical illness.ti,ab.
21	Ambulatory Care/
22	(urgent adj1 care).ti,ab.
23	Critical Care/
24	16 or 17 or 18 or 19 or 20 or 21 or 22 or 23
25	(community-dwelling or community dwelling).tw,kw.
26	9 and 12 and 15
27	limit 26 to (english language and yr="2010 - 2023")

28	("meta analysis" or meta-analysis or "meta synthesis" or meta-synthesis or "systematic review" or "review").ti,ab.
29	27 and 28
30	9 and 15 and 24
31	limit 30 to (english language and yr="2010 - 2023")
32	("meta analysis" or meta-analysis or "meta synthesis" or meta-synthesis or "systematic review" or "review").ti,ab.
33	31 and 32
34	9 and 15 and 25
35	limit 34 to (english language and yr="2010 - 2023")
36	("meta analysis" or meta-analysis or "meta synthesis" or meta-synthesis or "systematic review" or "review").ti,ab.
37	35 and 36
38	Costs.mp.
39	26 and 38
40	limit 39 to (english language and yr="2010 - 2023")
41	("meta analysis" or meta-analysis or "meta synthesis" or meta-synthesis or "systematic review" or "review").ti,ab.
42	34 and 38
43	40 and 41
44	limit 42 to (english language and yr="2010 - 2023")
45	("meta analysis" or meta-analysis or "meta synthesis" or meta-synthesis or "systematic review" or "review").ti,ab.
46	44 and 45
47	30 and 38
48	limit 47 to (english language and yr="2010 - 2023")
49	("meta analysis" or meta-analysis or "meta synthesis" or meta-synthesis or "systematic review" or "review").ti,ab.
50	48 and 49
51	health workforce/ or health personnel/ or health services/ or health staff/
52	26 and 51
53	limit 52 to (english language and yr="2010 - 2023")
54	("meta analysis" or meta-analysis or "meta synthesis" or meta-synthesis or "systematic review" or "review").ti,ab.
55	53 and 54
56	30 and 51
57	limit 56 to yr="2010 - 2023"
58	("meta analysis" or meta-analysis or "meta synthesis" or meta-synthesis or "systematic review" or "review").ti,ab.
59	57 and 58
60	34 and 51

61	limit 60 to (english language and yr="2010 - 2023")
62	("meta analysis" or meta-analysis or "meta synthesis" or meta-synthesis or "systematic review" or "review").ti,ab.
63	61 and 62

APA PsycInfo <1806 to June 2nd 2023>

Searches

1	Quality Indicators, Health Care.mp.
2	function*.mp. and exp Geriatric Assessment/
3	exp Program Evaluation/
4	(program* adj1 (evaluat* or effective*)).ti,ab.
5	(project adj1 (evaluat* or effective*)).ti,ab.
6	(quality or performance or process or outcome*).mp. [mp=title, abstract, heading word, table of contents, key concepts, original title, tests & measures, mesh]
7	indicator*.mp.
8	6 and 7
9	(quality or performance or process or outcome*).mp. [mp=title, abstract, heading word, table of contents, key concepts, original title, tests & measures, mesh]
10	assessment.mp.
11	9 and 10
12	bench marks.ti,ab.
13	1 or 2 or 3 or 4 or 5 or 8 or 11 or 12
14	exp Hospitalization/
15	Hip Fractures.mp.
16	exp Hospitals/ or exp Emergency Services/
17	Acute Care for Elders.ti,ab.
18	acute medical illness.ti,ab.
19	Ambulatory Care/
20	(urgent adj1 care).ti,ab.
21	Critical Care.mp.
22	14 or 15 or 16 or 17 or 18 or 19 or 20 or 21
23	exp Aging/ or aged.mp.
24	Seniors.ti,ab.
25	23 or 24

26	13 and 22 and 25
27	limit 26 to english language
28	limit 27 to yr="2010 -Current"
29	"meta analysis"/ or meta-analysis/ or "meta synthesis"/ or meat-synthesis.ti,ab.
30	review.ab,ti.
31	29 or 30
32	28 and 31
33	Long-Term Care/ or long term care/ or nursing homes/ or continuing care.ti,ab.
34	13 and 25 and 33
35	limit 34 to english language
36	limit 35 to yr="2010 -Current"
37	31 and 36
38	community-dwelling/ or community dwelling.mp. [mp=title, abstract, heading word, table of contents, key concepts, original title, tests & measures, mesh]
39	13 and 25 and 38
40	31 and 39
41	limit 40 to english language
42	limit 41 to yr="2010 -Current"
43	exp Health Care Costs/ or exp "Costs and Cost Analysis"/ or costs.mp.
44	32 and 43
45	37 and 43
46	42 and 43
47	health workforce/ or health personnel/ or health services/ or health staff/
48	32 and 47
49	37 and 47
50	42 and 47

Ebscohost CINAHL Plus with Full Text

#	Query	Limiters/Expanders
**S29**	S19 AND S23	Expanders - Apply equivalent subjects<br>Search modes - Find all my search terms
**S28**	S19 AND S22	Expanders - Apply equivalent subjects<br>Search modes - Find all my search terms

**S27**	S19 AND S21	Expanders - Apply equivalent subjects<br>Search modes - Find all my search terms
**S26**	S20 AND S23	Expanders - Apply equivalent subjects<br>Search modes - Find all my search terms
**S25**	S20 AND S22	Expanders - Apply equivalent subjects<br>Search modes - Find all my search terms
**S24**	S20 AND S21	Expanders - Apply equivalent subjects<br>Search modes - Find all my search terms
**S23**	S17 AND S18	Limiters - English Language; Published Date: 20100101-20230602; Publication Type: Meta Analysis, Meta Synthesis, Review, Systematic Review<br>Expanders - Apply equivalent subjects<br>Search modes - Find all my search terms
**S22**	S15 AND S18	Limiters - English Language; Published Date: 20100101-20230602; Publication Type: Meta Analysis, Meta Synthesis, Review, Systematic Review<br>Expanders - Apply equivalent subjects<br>Search modes - Find all my search terms
**S21**	S13 AND S18	Limiters - English Language; Published Date: 20100101-20230602; Publication Type: Review<br>Expanders - Apply equivalent subjects<br>Search modes - Find all my search terms
**S20**	costs or cost or expense or price or budget or financial	Limiters - English Language; Published Date: 20100101-20230602; Publication Type: Meta Analysis, Meta Synthesis, Review, Systematic Review<br>Expanders - Apply equivalent subjects<br>Search modes - Find all my search terms
**S19**	health workforce or health personnel or workforce or health staff or health care workers	Limiters - English Language; Published Date: 20100101-20230602; Publication Type: Meta Analysis, Meta Synthesis, Review, Systematic Review<br>Expanders - Apply equivalent subjects<br>Search modes - Find all my search terms
**S18**	aged or seniors	Expanders - Apply equivalent subjects<br>Search modes - Find all my search terms
**S17**	S11 AND S16	Limiters - English Language; Published Date: 20100101-20230602; Publication Type: Meta Analysis, Meta Synthesis, Review, Systematic Review<br>Expanders - Apply equivalent subjects<br>Search modes - Find all my search terms
**S16**	"community care" or "community nursing" or "primary care"	Expanders - Apply equivalent subjects<br>Search modes - Find all my search terms
**S15**	S11 AND S14	Limiters - English Language; Published Date: 20100101-20230602; Publication Type: Meta Analysis, Meta Synthesis, Review, Systematic Review<br>Expanders - Apply equivalent subjects<br>Search modes - Find all my search terms
**S14**	acute care setting or hospital or inpatient or hospitalization	Expanders - Apply equivalent subjects<br>Search modes - Find all my search terms
**S13**	S11 AND S12	Expanders - Apply equivalent subjects<br>Search modes - Find all my search terms

**S12**	long-term care or nursing home or continuing care or assisted living	Limiters - English Language; Published Date: 20100101-20230602; Publication Type: Review; Language: English<br>Expanders - Apply equivalent subjects<br>Search modes - Find all my search terms
**S11**	S1 OR S2 OR S3 OR S4 OR S5 OR S6 OR S7 OR S8 OR S9	Limiters - English Language; Published Date: 20100101-20230602; Publication Type: Review; Language: English<br>Expanders - Apply equivalent subjects<br>Search modes - Find all my search terms
**S10**	(S1 OR S2 OR S3 OR S4 OR S5 OR S6 OR S7 OR S8 OR S9)	Expanders - Apply equivalent subjects<br>Search modes - Find all my search terms
**S9**	quality indicators in healthcare	Expanders - Apply equivalent subjects<br>Search modes - Find all my search terms
**S8**	"Program* Evaluation"	Expanders - Apply equivalent subjects<br>Search modes - Boolean/Phrase
**S7**	TI (program* N3 (evaluat* or effective*)) OR AB (program* N3 (evaluat* or effective*))	Expanders - Apply equivalent subjects<br>Search modes - Boolean/Phrase
**S6**	TI "bench marks" OR AB "bench marks"	Expanders - Apply equivalent subjects<br>Search modes - Find all my search terms
**S5**	TI (((quality or performance or process or outcome*) and indicator*)) OR AB (((quality or performance or process or outcome*) and indicator*))	Expanders - Apply equivalent subjects<br>Search modes - Find all my search terms
**S4**	((quality or performance or process or outcome*) and indicator*)	Expanders - Apply equivalent subjects<br>Search modes - Find all my search terms
**S3**	TI (project N3 (evaluat* or effective*)) OR AB (project* N3 (evaluat* or effective*))	Expanders - Apply equivalent subjects<br>Search modes - Boolean/Phrase
**S2**	TI ((program* N3 (evaluat* or effective*)) OR TI ((program* N3 (evaluat* or effective*))	Expanders - Apply equivalent subjects<br>Search modes - Boolean/Phrase
**S1**	"Functional assessment"	Expanders - Also search within the full text of the articles<br>Search modes - Boolean/Phrase
