# Supplementary material for: Mapping Quality Indicators to Assess Older Adult Health and Care in Community-, Continuing-, and Acute-Care Settings: A Systematic Review of Reviews and Guidelines
Source: Healthcare (Basel). 2024 Jul 12;12(14):1397. doi: 10.3390/healthcare12141397 (PMC11276513; doi:10.3390/healthcare12141397)
Supplement: Supplementary file 1 [file healthcare-12-01397-s001.zip › Table S1. The methodological quality assessment of the included studies..pdf]

Table S1. The methodological quality assessment of the included studies.

First Author (Year)	1	2	3	4	5	6	7	8	9	10	11
**Amador, 2019**	Y	Y	Y	Y	Y	Y	Y	N/A	Y	Y	Y
**Askari, 2011**	Y	Y	Y	Y	N	N	Y	N/A	N	Y	Y
**Aitken, 2015**	Y	Y	Y	C	N	N	C	N/A	C	Y	Y
**Angel-Garcia, 2022**	Y	Y	Y	Y	N	N	Y	N/A	Y	Y	Y
**Arslan, 2021**	Y	Y	Y	Y	N	N	Y	N/A	Y	Y	Y
**Baldwin, 2017**	Y	Y	Y	Y	C	N	C	Y	C	Y	Y
**Burkett, 2017**	Y	Y	Y	Y	Y	C	Y	N/A	Y	Y	Y
**Chambers, 2023**	Y	Y	Y	Y	Y	Y	Y	N/A	Y	Y	Y
**Foong, 2022**	Y	Y	Y	Y	Y	Y	Y	N/A	C	Y	Y
**Giroux, 2023**	Y	Y	Y	Y	N	N	Y	N/A	N	Y	Y
**Griggs, 2020**	Y	Y	Y	Y	N	N	Y	N/A	Y	Y	Y
**Hillen, 2015**	Y	Y	Y	Y	Y	C	Y	N/A	Y	Y	Y
**Hutchinson, 2010**	Y	Y	Y	Y	Y	C	Y	N/A	Y	Y	Y
**Jajszczok, 2023**	Y	Y	Y	Y	N	N	Y	N/A	Y	Y	Y
**Jebeli, 2021**	Y	Y	Y	Y	N	N	Y	N/A	Y	Y	Y
**Joling, 2018**	Y	Y	Y	Y	Y	Y	Y	N/A	Y	Y	Y
**Lee, 2021**	Y	Y	Y	Y	Y	Y	Y	N/A	Y	Y	Y
**Lorini, 2018**	Y	Y	Y	C	N	N	C	N/A	C	Y	Y
**Mitchell, 2023**	Y	Y	Y	Y	Y	Y	Y	N/A	Y	Y	Y
**Osińska, 2022**	Y	Y	Y	Y	Y	Y	Y	N/A	Y	Y	Y
**Pitzul, 2017**	Y	Y	Y	Y	N	N	Y	N/A	C	C	Y
**Rand, 2021**	Y	Y	Y	C	N	N	C	N/A	C	Y	Y
**Schneberk, 2022**	Y	Y	Y	Y	Y	Y	Y	N/A	C	Y	Y
**Spilsbury, 2011**	Y	Y	Y	Y	C	C	Y	Y	Y	C	Y
**Tran, 2019**	Y	Y	C	C	C	C	Y	N/A	C	Y	Y
**Wagne, 2020**	Y	Y	Y	Y	Y	Y	Y	N/A	Y	C	Y
**Yorganci, 2021**	Y	Y	Y	Y	Y	Y	Y	N/A	Y	Y	Y

Y= Yes, N=No, N/A: Not applicable, C= Can not tell
